# The rules of scientific rigor: response to Shirzad and Abbassian

**DOI:** 10.1186/s12960-022-00717-0

**Published:** 2022-03-12

**Authors:** Jenny Carè, Amie Steel, Jon Wardle

**Affiliations:** 1 Australian Research Centre in Complementary and Integrative Medicine, Faculty of Health, University of Technology Sydney, Ultimo, Sydney, Australia; 2 National Centre for Naturopathic Medicine, Southern Cross University, Lismore, NSW Australia

We thank Shirzad and Abbassian for their response to our paper *‘Stakeholder attitudes to the regulation of traditional and complementary medicine professions: a systematic review’* [1] and for their comments regarding the importance of research in the area of regulation of traditional, complementary, and integrative medicine. The authors raise important issues regarding the need for comprehensive search strategies in systematic reviews.

Like Shirzad and Abbassian, we too believe that research should be rigorous and broad-ranging. Consequently, we included eight scientific databases in our search protocol as well as searching Google Scholar. We used between 37 and 53 traditional and complementary medicine search terms (including variations in terminology and spelling), and between 25 and 35 regulation-related terms (including variations), dependent on the specific database searched. As outlined in the Methods section of our paper, we consulted an authoritative source for a list of traditional and complementary medicine search terms; Cochrane Complementary Medicine [6].

While we agree that the traditional medicine systems suggested by Shirzad and Abbassian, namely, Persian medicine and Uyghur medicine, are important they do not appear in the Cochrane complementary medicine operational definition. Other traditional medicine systems are similarly not on the Cochrane list and were not included in the search protocol. Examples include Aboriginal traditional medicine, Bulgarian traditional medicine, and Samoan traditional medicine. This is not to say that these systems of medicine are not important or worthy of research. Rather, as Shirzad and Abbassian point out, when conducting research, investigators must follow the rules of scientific rigor, which includes providing a rationale for the choice of search terms. Our rationale was the use of the Cochrane list. Noting the importance of traditional medicine systems to the majority of the world’s population, we consider a review of the Cochrane complementary medicine operational definition may be required, with a focus on medicine systems less well-known to English-speaking audiences. An updated list that includes the previously mentioned medicine systems, as well as others, would greatly facilitate research into traditional, complementary, and integrative medicine.

The aim of our research was to investigate attitudes of a range of stakeholder groups towards the regulation of traditional and complementary medicine occupations. As well as investigating medicine systems that were not on the Cochrane list, the research cited by Shirzad and Abbassian, did not meet other criteria for inclusion in our research. The document analysis conducted by Negahban et al. [3] gave interesting insights into the existence of policies and laws regarding integration of traditional medicine in the Iranian health system, however, it did not encompass stakeholder attitudes to regulation. The second study referred to by Shirzad and Abbassian sought to develop a classification system for Persian medicine in Iran [5]. This study likewise did not canvass stakeholder attitudes towards occupational regulation. The final research study by Wusiman et al. [7] gives an engrossing account of Uyghur medicine which, while important, nonetheless does not meet the inclusion specifications of our systematic review.

As a matter of interest, upon your correspondence we undertook a further search of Ovid MEDLINE and PubMed using the regulation terms detailed in our paper with “Persian medicine” and Uyghur. No references were retrieved from Ovid MEDLINE and two unrelated articles were retrieved from PubMed.

Shirzad and Abbassian raise a valid point that further research effort is required in the field of traditional, complementary, and integrative medicine regulation. We agree that this is indeed worthwhile to identify existing studies, such as those cited by Shirzad and Abbassian, as well as explore new research areas in the field of occupational regulation. This consideration is particularly relevant given that the last World Health Organization Global Atlas on Traditional, Complementary and Alternative Medicine was almost two decades ago [4]. Research into unexplored areas of regulation of traditional, complementary, and integrative medicine is part of a body of work we hope to contribute to in other endeavours. One such research effort we have been involved in is the recently published World Naturopathic Federation Health Technology Assessment [2]. This 754 page book was compiled over 12 months by 51 naturopathic researchers globally, encompassing 2000 peer reviewed articles on the practice, efficacy, safety, and state of regulation of naturopathy worldwide. This and other research in traditional and complementary medicine involve considerable effort and are of immense importance to the field.

We thank Shirzad and Abbassian again for their interest in our work. It is heartening to see other research teams interested in the global regulatory and legislative landscape for traditional, complementary, and integrative medicine and we hope that this may present an opportunity for collaboration in the future in this area of mutual interest.

## Data Availability

Not applicable.
